# Levels, antecedents, and consequences of critical thinking among clinical nurses: a quantitative literature review

**DOI:** 10.3352/jeehp.2020.17.26

**Published:** 2020-09-07

**Authors:** Yongmi Lee, Younjae Oh

**Affiliations:** 1 College of Nursing, Kangwon National University, Chuncheon, Korea; 2 College of Nursing, Research Institute of Nursing Science, Hallym University, Chuncheon, Korea; Hallym University, Korea

**Keywords:** Nurses, Critical thinking, Literature review

## Abstract

The purpose of this study was to obtain a more comprehensive understanding of critical thinking within the clinical nursing context. In this review, we addressed the following specific research questions: what are the levels of critical thinking among clinical nurses?; what are the antecedents of critical thinking?; and what are the consequences of critical thinking? A narrative literature review was applied in this study. Thirteen articles published from July 2013 to December 2019 were appraised since the most recent scoping review on critical thinking among nurses was conducted from January 1999 to June 2013. The levels of critical thinking among clinical nurses were moderate or high. Regarding the antecedents of critical thinking, the influence of sociodemographic variables on critical thinking was inconsistent, with the exception that levels of critical thinking differed according to years of work experience. Finally, little research has been conducted on the consequences of critical thinking and related factors. The above findings highlight the levels, antecedents, and consequences of critical thinking among clinical nurses in various settings. Considering the significant association between years of work experience and critical thinking capability, it may be effective for organizations to deliver tailored education programs on critical thinking for nurses according to their years of work experience.

## Introduction

### Rationale

As the healthcare environment has become more complicated and detail-oriented and health professions have become more advanced, more nursing professionalism has been expected in recent years. To be more competent, nurses should be critical thinkers who can effectively cope with advancing technologies, human resource limitations, and the high level of acuity required in diverse healthcare settings. Critical thinking (CT) is considered to be a crucial element for clinical decision-making by nurses, and improved empowerment to engage in CT is considered to be a core program outcome in nursing education. However, recent studies have reported difficulties in applying CT to nursing practice [1,2], moderately low levels of CT among nurses [3], and differences in the understanding of the meaning of CT among nursing educators and scholars [4].

Since CT was emphasized as an essential component of the nursing process in the 1970s, numerous nursing scholars have attempted to define the concept of CT for nursing [5]. During the introductory period of CT, intellectual or cognitive skills were mostly emphasized. A decade later, affective disposition was also noted as an important component of CT in the context of a caring relationship [6]. Emotional involvement enables nurses to genuinely feel the suffering and pain that patients experience [7]. In 2000, Scheffer and Rubenfeld [8] identified essential components of CT, including 10 affective habits of the mind and 7 cognitive skills, by using the Delphi method to arrive at a consensus on an acceptable definition of CT. In recent years, nurses have been increasingly expected to develop both CT affective dispositions and CT cognitive skills [9]. Affective dispositions such as being open-minded, inquisitive, and seeking truth can stimulate an individual towards using CT through a reasoning process [10]. Meanwhile, cognitive skills may help nurses analyze their inferences, explain their interpretations, and evaluate their analyses [11]. Knowledge is also necessary to strengthen and support the cognitive process of CT [3,10].

To our knowledge, the most recent scoping review on the concept of CT in the nursing field was reported in 2015 [5]; according to this comprehensive review [5], there was growing interest in the study of the concepts and dimensions of CT experienced by nurses and nursing students, as well as in the development of training strategies for both students and professionals. However, a direction for further research into CT among clinical nurses was to specifically focus on its features or tendencies and changes in the CT phenomenon as time goes by, because confusing perspectives and poor knowledge of CT among nurse-educators can threaten the nursing profession [12]. Furthermore, an extensive review of quantitative research findings on CT among nurses is lacking, since only a scoping review was published in 2015 [5]. Thus, it is necessary to better understand how clinical nurses exercise CT to cultivate their clinical decision-making skills by reflecting on the contemporary nursing context.

### Objectives

The purpose of this study was to obtain a more comprehensive understanding of CT in the clinical nursing context. In this review, we specifically addressed the following research questions: what are the levels of CT among clinical nurses?; what are the antecedents of CT?; and, what are the consequences of CT?

## Methods

### Ethics statement

This study did not have human subjects; therefore, neither institutional review board approval nor informed consent was required.

### Study design

A narrative literature review was used. We followed the methodologies described by the Center for Reviews and Dissemination for undertaking reviews [13] and by Petticrew and Roberts [14], who addressed the practical guide as an alternative to systematic reviews in the social sciences, since our major goal was to synthesize the individual studies narratively and not to evaluate the efficacy and safety of interventions or programs.

### Materials and/or subjects

#### Information sources

In this study, CT in clinical nursing was analyzed using a narrative review design to provide an overview of CT among nurses. We conducted an extensive search in the MEDLINE, Cumulative Index to Nursing and Allied Health Literature (CINAHL), and Ovid databases for articles published from July 2013 to December 2019 on CT among nurses, since Zuriguel Pérez et al. [5] comprehensively conducted a scoping review of articles on this topic that included research published from January 1999 to June 2013.

#### Search

The following keywords were used: “critical thinking,” “professional judgment,” “clinical judgment,” and “clinical competence.” We also used the snowball method to identify additional studies. Titles and abstracts were screened, and studies were included if they presented empirical research on CT among clinical nurses, published in English from July 2013 to December 2019. Publications were excluded if they were reviews, case studies, or unpublished dissertations, or if CT was only studied among nursing students.

### Search outcomes

#### Phase 1

**Fig. 1.**
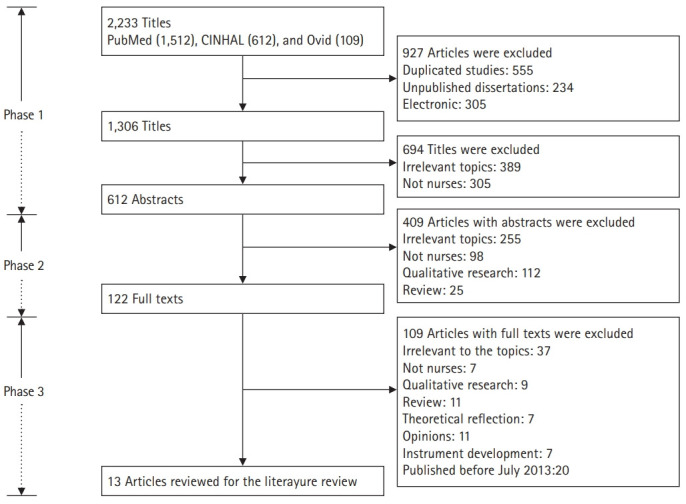
Flow diagram of the process of identifying and including articles for this review.

Both researchers (Y.L. and Y.O.) carried out the literature search to ensure that all relevant articles would be identified. The search produced a total of 2,233 articles. Candidate articles were screened by title. Titles that both researchers agreed were irrelevant to the aim of this review, as well as duplicates, were excluded. All other articles (612) were assessed as potentially relevant to the topic, and those for which consensus was reached between the authors were forwarded to the next phase (Fig. 1).

#### Phase 2

All abstracts from the articles selected during phase 1 were evaluated by reading them and checking whether they met the inclusion criteria. All studies that met the criteria proceeded to the next phase of the search process. If no consensus was reached for a particular article, the article was also forwarded to the next phase. All other studies (490) were excluded (Fig. 1).

#### Phase 3

In the final phase of the search process, a total of 122 articles from phase 2 were read and evaluated in light of the inclusion criteria. Of these, articles with text irrelevant to the study (109) were excluded, as the papers did not focus on CT or nurses, did not employ quantitative research design, or were published before July 2013. A final number of 13 articles were included (Fig. 1).

### Quality appraisal

We evaluated the included studies using assessment sheets prepared and tested by Hawker et al. [15], who developed an instrument that is capable of appraising methodologically heterogeneous studies. The data extraction sheet explores 9 components in detail: title and abstract, introduction and aims, method and data, sampling, data analysis, ethics and bias, results, transferability or generalizability, and implications and usefulness. In our review, each of these areas was assessed using the criteria developed by Hawker et al. [15] and rated on a scale of 1 (very poor) to 4 (very good). The scores for each assessment were then summed to obtain an overall score and rating, which ranged from very poor (9) to very good (36). Any article scoring less than 18 was considered to be of poor to very poor quality.

Using the assessment described above, the selected studies had scores ranging from 23 to 33 out of 36. Hence, all studies were included in the review (Table 1). All studies mentioned either a research question or an objective. In each article, the study design was described. Procedures or interventions were described in all studies, including three quasi-experimental studies [16-18]. Random sampling and purposive sampling were most commonly employed. Some studies, however, failed to state their sampling methods. Five studies determined the sample size by using power analysis [16,19,20], the Raosoft sample size calculator [21], or Solvin’s formula [22]. All studies addressed ethical considerations except for 1 study [23]; however, no studies described or elaborated on whether the researchers had received permission to use an original or translated version of the research instruments.

### Data abstraction and synthesis

The data abstraction and synthesis process consisted of re-reading, isolating, comparing, categorizing, and relating relevant data. Included articles were read repeatedly to obtain an overall understanding of the material. Relevant data were gathered and classified into 3 categories: levels of CT, antecedents of CT, and consequences of CT.

## Results

### Study selection

Our review included 13 publications (Table 1). The studies were conducted in 7 different countries: Korea and the United States (n=3, for each country), Spain and Taiwan (n=2, for each country); and Malaysia, Turkey, and Egypt (n=1, for each country). The research settings were hospitals (n=6), intensive or critical care units (n=3), acute care units (n=2), and psychiatric care units (n=1). One study included nurses working in critical care and emergency units [24]. In all studies, the sample consisted of only nurses.

### Study characteristics

The methodological features of the included studies are summarized in Table 1. Twelve studies implemented quantitative research to examine the phenomenon of CT, while 1 study used a mixed-methods research approach [18]. Six of the included studies implemented a quasi-experimental design to evaluate the effect of their programs on CT among clinical nurses [16-18,23-25]. All included studies described institutional review board approval, except for 3 studies, which either stated that researchers verbally obtained the consent of the participants to be included in the research [22,26] or did not mention this issue [23].

CT was evaluated employing the California Critical Thinking Disposition Inventory [21,22,24,26], Critical Thinking Disposition Inventory [18,19], Critical Thinking Disposition [17,20], Nursing Critical Thinking in Clinical Practice Questionnaire [27,28], Clinical Critical Thinking Skill Test [16], Health Sciences Reasoning Test (HSRT) [25], or a self-evaluation tool to measure 5 key indicators of the development of CT [23]. All studies, except for 1, utilized a validated version of the original instruments in the appropriate language or validated the instruments in their research [22]. All studies except for 3 reported internal consistency reliability [22,23,25].

### Levels of critical thinking

All studies measured the levels of CT among nurses, except for 1 study [20] (Table 1). Clinical nurses in 4 studies reported low [26], moderate [22,27], and high [21] levels of CT. Chen et al. [19] reported that experienced nurses, with an average of 18.38 years of work experience, had higher CT scores than novice registered nurses did. Similarly, Zuriguel-Perez et al. [28] reported that the level of CT among more experienced nurse managers was higher than among other nurses.

Five studies showed that their developed programs significantly improved the levels of CT among nurses in the experimental group compared to nurses in the control group [16-18,23,24]. One study presented a significant increase in the mean overall CT score for the HSRT on the posttest using a 1-group pretest-posttest design [25]. In particular, 5 programs—a work-based critical reflection program [16], a scenario-based simulation training program [17], case studies with videotaped vignettes [25], and concept mapping [23]—had positive effects on CT levels among novice nurses. Zori et al. [24] reported significant effects of a reflective journaling exercise to strengthen CT dispositions among nurses with diverse work experience. Hung et al. [18] developed a problem-based learning program for mental health care nurses for 3 hours every week, for a total of 5 weeks (15 hours total).

### Antecedents of critical thinking

Seven studies reported inconsistent findings regarding the influence of 1 or more sociodemographic variables on CT (Table 1). According to these studies, there were significant differences in CT across sociodemographic variables, including age [19,21,27,28], gender [21], ethnicity [21], years of experience [19,21,27,28], and educational level [21,28]. In 3 studies, older nurses, those with more clinical experience, or those with higher levels of education had higher levels of CT [19,21,28]. Although Ludin [21] reported significant differences in the levels of CT according to gender, ethnicity, and educational level, detailed information was not provided. In contrast, Mahmoud and Mohamed [22] reported that none of the sociodemographic variables or job characteristics had statistically significant relationships with the total CT disposition and, in 2 studies, there were no significant relationships between CT levels and educational level [26,27], years of experience [26], gender [27], or work units [27]. Nurses had higher levels of CT when they had higher levels of self-reflection [19] and lower levels of perception of barriers to research use [20].

### Consequences of critical thinking

Only 1 study investigated the consequences of CT [20], and found that CT disposition of nurses positively influenced evidence-based practice (Table 1). In this study, Kim et al. [20] found that the relationship between barriers to research use and evidence-based practice was mediated by CT disposition.

## Discussion

### Methodological issues

Studies from 6 different countries were included. Most of the studies were done in Asian countries; only 2 of the studies were conducted in Europe. Synthesizing and integrating data from different countries and cultures is a complex and challenging task [29], especially since differences in cultural attitudes on CT extend beyond our expertise. The restricted professional autonomy perceived by nurses, which impeded CT, may be different for each culture or country. For instance, several studies in Asia have reported that nurses lack or have limited authority in providing care for their patients [30,31]; furthermore, while CT allows nurses to generate new ideas quickly, become more flexible, and act independently and confidently, the scope of their action is still ultimately limited by the physician’s clinical decisions [10]. Thus, several Asian nursing scholars have stressed the growth of professional autonomy among nurses through exercising higher levels of CT as an area that needs support to improve nurses’ clinical competence.

In the studies we reviewed, 7 different instruments were used. Due to this diversity of instruments, it was difficult to compare and integrate quantitative data. In addition, some studies utilized instruments without testing reliability and validity; thus, it is recommended to validate CT assessment instruments used in future research to ensure their reliability. A large range in sample sizes and response rates, possible non-responder bias, and validation of the instruments restricted to small populations limited the representativeness of our study results.

### Substantive findings

Although the assessment tools used to measure the level of CT varied across the studies reviewed, the level of CT was mostly moderate or high among the nurses evaluated. This may be partly due to the emphasis of CT in nursing education in recent years; furthermore, CT is now recognized as an essential competency among nurses and is required for the accreditation of nursing education [9,32]. However, this result contrasts with other research that reported a low level of CT among nursing students [33]. Further research is needed to verify the differences between nurses and nursing students according to factors influencing CT disposition and skills.

Our review complements the results of a previous review that scoped the concept of CT in the nursing field [5]. For instance, as antecedents of CT, the association between sociodemographic variables and CT can only be revealed by quantitative studies. It is necessary to examine the relationships between them in the future since the influence of sociodemographic variables on CT was found to be inconsistent in our study, except for years of work experience, which showed a consistent association with CT capacity. This finding may be associated with the significant experience gained by more senior nurses, which complements their theoretical knowledge and clinical decision-making [34] and enables them to be capable of better reflecting on past experiences, which may foster a deeper understanding of the situation [19]. On the contrary, less-experienced nurses had difficulties in exercising CT because of their perceptions of a gap between theory and practice with reference to their education and the real workplace setting [16]. Thus, it can be useful for senior nurses to share and reflect on their successful experiences of applying CT for patient care through group discussions; meanwhile, for novice nurses, a clear and detailed approach on exercising CT to reduce the gap between theories and the clinical setting may be beneficial. For this reason, a tailored education program on CT should be developed according to nurses’ years of work experience.

Self-reflection was also significantly related to CT among nurses in our review. This finding can be explained in terms of genuine self-reflection which can help them develop their CT dispositions and skills by balancing a lack of confidence and professional autonomy [34]. CT encourages nurses to generate new ideas quickly, be flexible, and act independently and confidently [29]. In contrast, nurses’ CT becomes more limited when they are more dependent on physicians’ clinical decisions. Meanwhile, expert nurses are not confined to or constrained by theoretical knowledge and are able to interpret situations by actively utilizing their nursing care experiences with past patients through exercising CT in their decision-making process [9,40]. To promote and encourage CT, nurses need to be more independent, confident, and responsible. As nurses’ autonomy develops, the need to think critically is further promoted [29,33]. However, nurses in some nursing environments have reported limited or restricted professional autonomy due to existing rigid and hierarchical cultures, as well as physician-centered paradigms in hospitals, which can hinder nurses from exercising CT [35-38]. More research is required regarding autonomy and CT among nurses in relation to their perceptions of the organizational atmosphere.

Our review revealed that there is limited empirical research on the consequences of CT, since only 1 of the included studies investigated the consequences of CT. CT stimulates nurses to explore related knowledge and establish priorities for solving patients` clinical problems [39]. As a method of assessing, planning, implementing, reevaluating, and reconstructing nursing care, a CT approach encourages nurses to challenge established theory and practice [5,9]. In addition, good clinical judgement results from exercising CT by advancing nursing competence in contemporary healthcare environments, where the complexity of data and amount of newly developed knowledge increases daily [10]. Papathanasiou et al. [9] emphasized that nurses’ ability to find specific solutions to certain problems is easily achieved when creativity and CT work together. Nevertheless, an integrated review found no relationship between CT and clinical decision-making in nursing [40]. Further research is recommended to explore the consequences of CT in nursing.

### Limitations

Despite complementing the findings of a previous scoping review [5], our review has 2 major limitations. First, a more synthesized approach should be attempted, including both quantitative and qualitative studies, in order to facilitate a more in-depth examination of our research topic. Second, access to all resources via electronic databases was not possible and only studies written in English were included in our review.

## Conclusion

Our review highlighted the levels, antecedents, and consequences of CT among clinical nurses in various settings. Further quantitative studies are recommended using representative sample sizes and validated instruments with high and stable reliability to enhance our knowledge of this issue through optimal methodologies. Considering the significant association between years of work experience and CT capability, it would be helpful and effective for organizations to deliver a tailored education program on CT developed according to years of work experience to enhance CT among nurses when providing care for their patients. To make progress towards this goal, however, further research is needed to clarify the antecedents of CT and to explore its consequences.

## Figures and Tables

**Table 1.** Studies included in the literature review

No.	Author (year)	Country/care setting(s)	Aim(s) of the study/research problem(s)	Design and sample/RR	Data collection/analysis	Ethical considerations
1	Chen et al. [19] (2019)	Taiwan; 1 medical center	To examine whether professional qualifications (e.g., age, years of job experience, and position on the clinical ladder) would affect self-reflection and CT in experienced RNs; levels and antecedents of CT	Quantitative research; descriptive and correlational; power analysis (effect size=0.3); 597 nurses (297 novice RNs, 300 experienced RNs); RR=96%	Questionnaires were collected in each ward box; questionnaires included: demographic data, Taiwan Critical Thinking Disposition Inventory, and Self-Reflection and Insight Scale; partial least squares structural equation modeling	Approval of the IRB
2	Zuriguel-Perez et al. [27] (2019)	Spain; medical, surgical, and critical care units at a tertiary care hospital	To identify the level of CT among nurses in clinical practice according to sociodemographic and professional variables; levels and antecedents of CT	Quantitative research; a descriptive cross-sectional and correlational study; 339 nurses	Questionnaires included: demographic data and Nursing Critical Thinking in Clinical Practice Questionnaire; Mann-Whitney U-test and Kruskal-Wallis H-test	Approval of the Clinical Research Ethics Committee of the Hospital Vall d’Hebron Hospital
3	Kim et al. [16] (2018)	Korea; 1 advanced general hospital	To evaluate the effectiveness of a work-based critical reflection program to enhance novice nurses’ clinical CT abilities, communication competency, and job performance; levels of CT; differences between experimental and control groups	Quantitative research; quasi-experimental design; power analysis (effect size=0.5); experimental group (24 novice nurses) and control group (20 novice nurses)	Questionnaires included: demographic data, Clinical Critical Thinking Skill Test, Global Interpersonal Communication Competency Scale, and performance measurement scale; non-parametric Mann-Whitney U-test and the Wilcoxon rank-sum test	Approval of the IRB; informed consent obtained; anonymity and confidentiality assured
4	Ludin [21] (2018)	Malaysia; 7 critical care environments in hospitals	To understand whether critical care nurses’ CT disposition affects their clinical decision-making skills; levels and antecedents of CT	Quantitative research; cross-sectional study, descriptive; purposive sample; Raosoft sample size calculator; 113 nurses	Questionnaires included: demographic data, Malay/English translation of the Short Form-Critical Thinking Disposition Inventory-Chinese version, and the Clinical Decision-making Nursing Scale; Pearson coefficient correlations; 1-way analysis of variance	Approval of the IRB; anonymity and confidentiality assured
5	Zuriguel-Perez et al. [28] (2018)	Spain; 1 tertiary hospital with 3 centers	To analyze the levels of CT among nurse managers and registered nurses and to explore the association between these levels and socio-demographic and occupational factors; levels and antecedents of CT	Quantitative research; cross-sectional study; random sample; 44 nurse managers and 295 RNs; RR=100% (nurse managers), RR=98.3% (RNs)	Questionnaires were distributed to nurses in person; questionnaires included: demographic data, Nursing Critical Thinking in Clinical Practice Questionnaire; multivariate analysis	Approval of the Clinical Research Ethics Committee of the Hospital Vall d’Hebron Hospital; informed consent obtained; anonymity and confidentiality assured
6	Jung et al. [17] (2017)	Korea; internal medicine from 4 university hospitals	To develop and test the effects of a scenario-based simulation training program on new graduate nurses' competency, CT dispositions, and interpersonal communication skills; levels of CT	Quantitative research; quasi-experimental design; experimental group (24 new graduate nurses) and control group (24 new graduate nurses)	Questionnaires included: demographic data, Holistic Nursing Competence Scale, Critical Thinking Disposition, and Interpersonal Communication Competence Scale; Mann-Whitney U-test	Approval of the Ethics Review Board
7	Mahmoud and Mohamed [22] (2017)	Egypt; 3 public hospitals	To investigate CT disposition among nurses working in public hospitals in the Port-Said Governorate; levels and antecedents of CT	Quantitative research; descriptive study; random sample; sample size was calculated by Slovin’s formula; 196 nurses	Questionnaires included: demographic data and California Critical Thinking Disposition Inventory; Kolmogorov-Smirnov test per sample value amounted to 0.939, exceeding the significance level of 0.341 that proved the normality of the variable	Verbal consent obtained; confidentiality assured
8	Yurdanur [26] (2016)	Turkey; intensive care units in a public hospital	To describe CT dispositions among critical care nurses in Turkey, and to study whether background data had any impact on CT dispositions; level and antecedents of CT	Quantitative research; descriptive study; 85 nurses; RR=81%	Face-to-face meetings with the nurses; questionnaires included: demographic data and the California Critical Thinking Disposition Inventory	Permission obtained from the institution where the research would take place; verbal consent obtained
9	Kim et al. [20] (2015)	Korea; acute care units of 4 university hospitals	To examine whether CT mediates the relationship between perceived barriers to research use and evidence-based practice in clinical nurses; antecedents and consequences of CT	Quantitative research; cross-sectional study; power analysis (effect size=0.3)	420 Questionnaires were mailed to 4 hospitals with instructions to place the completed survey in the provided envelope and to seal it; questionnaires included: demographic data, Evidence-Based Practice Questionnaire, Critical Thinking Disposition, and Barriers or Facilitators to Using Research in Practice Scale; Pearson’s correlation coefficients; structural equation modeling	Approval of the IRBs; informed consent obtained
				The sample size was calculated by a theoretical model (n≥200) and population model in structural equation modeling (n≥300); 409 RNs; RR=97.4%		
10	Hung et al. [18] (2015)	Taiwan; 1 Taipei city mental healthcare hospital	To share the use of this innovative strategy in continuing education and to examine the effectiveness of problem-based learning on CT; level and antecedents of CT; differences between experimental and control groups	Mixed methods research; quasi-experimental design; purposive sample; randomly allocated to each group; experimental group (22 nurses) and control group (22 nurses)	Questionnaires included: demographic data and Critical Thinking Disposition Inventory; open-ended interview with participants in the experimental group after 5 weeks of a problem-based learning program; quantitative data analysis: paired and unpaired t-test; qualitative data analysis: Colaizzi’s analysis	Approval of the IRB; confidentiality assured
11	Hooper [25] (2014)	USA; 1 acute care hospital	To determine if using case studies with videotaped vignettes helped facilitate the development of CT skills in new graduate nurses participating in a nurse residency program	Quasi-experimental design; convenience sample; one group pretest-posttest design; 18 new graduate nurses	Questionnaires included: demographic data and Health Sciences Reasoning Test; paired t-test	Approval of the IRB
12	Wahl and Thompson [23] (2013)	USA; critical care units	To evaluate the effectiveness of concept mapping as a teaching tool to improve CT and clinical decision-making skills in novice nurses	Quasi-experimental design; convenience sample; one group pretest-posttest design; 31 new graduate nurses	Questionnaires included: self-evaluation tool to measure 5 key indicators of the development of CT: problem recognition, clinical decision-making, prioritization, clinical implementation, and reflection; 1-tailed t-test	Not mentioned
13	Zori et al. [24] (2013)	USA; critical care and emergency units	To determine whether a reflective journaling exercise would strengthen the CT dispositions of participants in an RN fellowship program that was designed to transition nurses to practice in critical care and the emergency department	Quasi-experimental design; convenience sample; experimental group (53 nurses) and control group (62 nurses)	Questionnaires included: demographic data and California Critical Thinking Disposition Inventory; t-test; repeated-measures analysis of variance	Approval of the IRB

RR, response rate; CT, critical thinking; RN, registered nurse; IRB, institutional review board.

## Supplementary materials

Supplement 1. Audio recording of the abstract.
